# Interactive “Video Doctor” Counseling Reduces Drug and Sexual Risk Behaviors among HIV-Positive Patients in Diverse Outpatient Settings

**DOI:** 10.1371/journal.pone.0001988

**Published:** 2008-04-23

**Authors:** Paul Gilbert, Daniel Ciccarone, Stuart A. Gansky, David R. Bangsberg, Kathleen Clanon, Stephen J. McPhee, Sophia H. Calderón, Alyssa Bogetz, Barbara Gerbert

**Affiliations:** 1 Division of Behavioral Sciences, Professionalism, and Ethics, University of California San Francisco, San Francisco, California, United States of America; 2 Department of Family and Community Medicine, University of California San Francisco, San Francisco, California, United States of America; 3 Department of Anthropology, History and Social Medicine, University of California San Francisco, San Francisco, California, United States of America; 4 Division of Oral Epidemiology and Dental Public Health, University of California San Francisco, San Francisco, California, United States of America; 5 Epidemiology and Prevention Interventions (EPI) Center, Division of Infectious Diseases and Positive Health Program, San Francisco General Hospital, University of California San Francisco, San Francisco, California, United States of America; 6 East Bay AIDS Education and Training Center, Oakland, California, United States of America; 7 Division of General Internal Medicine, Department of Medicine, University of California San Francisco, San Francisco, California, United States of America; University of Cape Town, South Africa

## Abstract

**Background:**

Reducing substance use and unprotected sex by HIV-positive persons improves individual health status while decreasing the risk of HIV transmission. Despite recommendations that health care providers screen and counsel their HIV-positive patients for ongoing behavioral risks, it is unknown how to best provide “prevention with positives” in clinical settings. *Positive Choice*, an interactive, patient-tailored computer program, was developed in the United States to improve clinic-based assessment and counseling for risky behaviors.

**Methodology and Findings:**

We conducted a parallel groups randomized controlled trial (December 2003–September 2006) at 5 San Francisco area outpatient HIV clinics. Eligible patients (HIV-positive English-speaking adults) completed an in-depth computerized risk assessment. Participants reporting substance use or sexual risks (n = 476) were randomized in stratified blocks. The intervention group received tailored risk-reduction counseling from a “Video Doctor” via laptop computer and a printed Educational Worksheet; providers received a Cueing Sheet on reported risks. Compared with control, fewer intervention participants reported continuing illicit drug use (RR 0.81, 95% CI: 0.689, 0.957, p = 0.014 at 3 months; and RR 0.65, 95% CI: 0.540, 0.785, p<0.001 at 6 months) and unprotected sex (RR 0.88, 95% CI: 0.773, 0.993, p = 0.039 at 3 months; and RR 0.80, 95% CI: 0.686, 0.941, p = 0.007 at 6 months). Intervention participants reported fewer mean days of ongoing illicit drug use (-4.0 days vs. -1.3 days, p = 0.346, at 3 months; and -4.7 days vs. -0.7 days, p = 0.130, at 6 months) than did controls, and had fewer casual sex partners at (−2.3 vs. −1.4, p = 0.461, at 3 months; and −2.7 vs. −0.6, p = 0.042, at 6 months).

**Conclusions:**

The *Positive Choice* intervention achieved significant cessation of illicit drug use and unprotected sex at the group-level, and modest individual-level reductions in days of ongoing drug use and number of casual sex partners compared with the control group. *Positive Choice*, including Video Doctor counseling, is an efficacious and appropriate adjunct to risk-reduction efforts in outpatient settings, and holds promise as a public health HIV intervention.

**Trial Registration:**

Clinicaltrials.gov NCT00447707

## Introduction

Advances in HIV treatment have dramatically increased patients' duration and quality of life [1], [2]. Because HIV-positive individuals are living longer and may continue to engage in risky behaviors, new prevention strategies now address the role of HIV-positive persons; an approach called “prevention with positives.” To specifically address prevention with HIV-positive individuals, the US Centers for Disease Control (CDC) recommends that providers screen for and intervene on transmission-related risk behaviors, monitor behaviors that increase the risk of disease progression, and counsel patients on how they can protect their own health [3].

Reducing or eliminating unprotected sex has traditionally been a cornerstone of HIV prevention in the US [4]. In addition to the risk of HIV transmission, unprotected sex may adversely affect the HIV-positive individual's health. New sexually transmitted infections may exacerbate discomfort, and can increase viral load and accelerate disease progression [5], [6].

Reducing substance use is another important strategy to reduce transmission and protect the health of HIV-positive individuals [7], [8]. Both illicit drug and excessive alcohol use are associated with high-risk sexual behaviors [9]–[11]. Substance use is also a predictor of incomplete adherence to antiretroviral therapy [6], which in turn may lead to drug resistance [12], [13], as well as to more rapid progression to AIDS and mortality [14]–[16]. Consequently, reducing sexual risk and substance use can be reframed as lifestyle changes supporting the patient's own health while avoiding conceptualizing the HIV-infected person as a vector of disease [17].

Although the CDC recommends that providers screen and counsel HIV-infected patients for ongoing behavioral risks [3], many do not. Physicians' risk-reduction efforts are frequently constrained by discomfort with the topics, pessimism about patients' behavior change, confusion about their role as counselor, lack of time, and lack of confidence in their skills [18]–[21]. Nevertheless, a growing body of evidence suggests that interventions for HIV-positive individuals can reduce risky behaviors [22], and brief motivational interventions decrease unprotected sex [23], reduce harmful alcohol use [24], and increase adherence to antiretroviral therapy [25].

Despite increasing interventions designed to reduce risky behaviors, few focus specifically on HIV-positive individuals [26], address multiple risky behaviors [26], [27], or target more than 1 vulnerable population [27]. A recent review of the literature by Lyles et al. (2007) found only 18 interventions that met the criteria for a best-evidence HIV behavioral intervention as determined by the CDC's HIV/AIDS Prevention Research Synthesis Team. Of these, only 4 targeted HIV-positive individuals, with 1 exclusively for women and 1 exclusively for men who have sex with men. These interventions also demanded substantial time commitments from both patients and health care providers. Considering the existing constraints on physicians' risk screening and counseling practices [28], these interventions are less than ideal for clinical practice settings.

Efficacious prevention interventions that can be seamlessly integrated into HIV clinical settings are essential to address ongoing sensitive risk behaviors. The Center for Health Improvement and Prevention Studies (CHIPS) has adapted multimedia computer technology to support these efforts, creating a computer program that involves both patients and providers [29]. Delivered on laptop computers in clinic settings, this computer program conducts in-depth risk assessments, delivers tailored counseling messages, and produces printed output for both the patient and provider. A novel component is the “Video Doctor” intervention, an actor-portrayed physician who engages patients in a confidential, “face-to-face” discussion about risky behavior. The Video Doctor simulates an ideal conversation with a health care provider, and has been highly acceptable to diverse primary care patients in the US [29].

Using a Video Doctor counselor and a framework that emphasized concern for the patient's own health rather than solely transmission of HIV [17], we developed *Positive Choice*, an interactive computer program to improve screening and counseling about ongoing risky behaviors in HIV-infected patients. We conducted a randomized, controlled trial of *Positive Choice* to test its efficacy at reducing illicit drug use, risky alcohol drinking, and anal or vaginal intercourse without a condom.

## Methods

The protocol for this trial and supporting CONSORT checklist are available as supporting information; see Checklist S1 and Protocol S1.

### Sample size, sites, and recruitment

A series of power analyses were calculated for 2 group comparisons of each risk as a binary outcome (any ongoing risk vs. none) with at least 80% power to detect a difference in proportions of at least 0.125 with the control group proportion in the range 0.10 to 0.90 (2 tailed, α = 0.05), resulting in a target sample size of 526 participants.

Between December 2003 and September 2006 the *Positive Choice* trial was integrated into 5 outpatient HIV clinics in the San Francisco Bay Area, including 2 public hospitals, a community-based organization, a private hospital, and a health maintenance organization (HMO). Eligible participants were age 18 or older and HIV-positive 3 months or longer. *Positive Choice* was available in English only. Participants were recruited via clinic advertisements (posters and flyers) and self-referred or were referred by clinic staff or providers. Four sites allowed direct recruitment by research assistants, who serially approached patients in waiting rooms as they arrived for scheduled appointments. If patients indicated an interest in participating, research assistants escorted them to a private area of the clinic where eligibility criteria were assessed in a structured in-person interview. All participants provided informed consent and received a $40 gift card as compensation for completing a baseline session. Compensation increased to $50 and $60 at 3- and 6-month follow-ups. Retention was maximized using frequent reminders by phone or mail. Study procedures were approved by the University of California San Francisco's Committee on Human Research.

### Risk assessment and randomization

Participants used a laptop computer to complete the *Positive Choice* risk assessment, a low-literacy-demand computerized interview with audio voiceover. Privacy was assured by use of a private examination room and headphones. All baseline and follow-up risk assessments were done approximately 1 hour prior to a regularly scheduled medical appointment, allowing participants ample time to complete the computer session before the scheduled medical appointment. In this way, *Positive Choice* was integrated into the flow of each clinic.

The assessment collected self-reported demographic information (including race/ethnicity), baseline clinical information (such as length of time HIV-positive), and screened participants for drug, alcohol, and sexual risks. The assessment captured days of use in the past month for 10 illicit drugs. Drug risk was defined as 1) any use of the following: crack cocaine; methamphetamine; powder cocaine; “speedball” (heroin with cocaine); or heroin; or 2) 3 or more days of use of the following: “downers” (e.g., barbiturates); non-prescribed opiates; inhalants; hallucinogens; and “ecstasy” (methylenedioxymethamphetamine [MDMA]). To avoid contradicting messages about purported medicinal use, marijuana use was not categorized as a drug risk. Risky alcohol use was defined as exceeding the US National Institute on Alcoholism and Alcohol Abuse's recommended number of drinks per week (14 or fewer for men; 7 or fewer for women) and/or 3 or more binge drinking episodes (5 or more drinks on 1 occasion for men; 4 or more drinks on 1 occasion for women) within the previous 3 months.

Sexual risk was defined as anal or vaginal intercourse without a condom; the program did not inquire about oral sex. Participants were asked for the total number of sex partners in the last 3 months, then asked to report condom use as a numeric percentage, from 0% (never used) to 100% (consistently used), with a main partner and/or up to 5 casual partners in the previous 3 months. Sexual risk was operationalized as a dichotomous variable (100% condom use versus <100%) with main and/or casual partners, yielding a conservative definition of sexual risk. Participants were also asked about their sex partners' HIV status (HIV-negative, HIV-positive, or unknown) to allow further tailoring of intervention messages; partners' HIV status did not determine sexual risk status.

Randomization by the computer occurred immediately upon completion of the baseline risk assessment and was independent of the research assistants. Participants reporting 1 or more risky behaviors were stratified by risk profile (drug risk; alcohol risk; sex risk; drug and alcohol risks; drug and sex risks; alcohol and sex risks; drug, alcohol, and sex risks) then assigned to intervention or control groups in blocks of 1, resulting in equivalent intervention and control groups for each risk combination. Patients and their providers were not informed of group assignment, although assignment to the intervention group might have been deduced by some patients and their providers by receipt of printed output (Educational Worksheet and Cueing Sheet, respectively) from the computer. Research assistants were aware of group assignment only upon completion of the patient's baseline session. Both intervention and control participants completed follow-up risk assessments 3 and 6 months post-baseline.

### Video Doctor intervention

Upon completion of the risk assessment, the *Positive Choice* program immediately played the Video Doctor clips for participants randomized to the intervention group, thus creating a seamless transition to the intervention segment. Interactive risk-reduction messages, based on principles of Motivational Interviewing [30], [31], were delivered by an actor-portrayed Video Doctor, whose tone was respectful and non-judgmental. These messages simulated an ideal discussion where the health care provider expressed reflexive understanding of the patient's concerns, showed compassion for the patient, and provided non-judgmental counseling. We did not expect that a computer program could replace a skilled counselor; but somewhat counter-intuitively, computer technology may actually help increase fidelity to some principles of Motivational Interviewing (MI). Correct implementation of MI is highly dependent on an individual's counseling skills and abilities. By standardizing messages and using complex, interactive programming to tailor responses, our program was able to construct a seamless counseling session with the Video Doctor closely adhering to several key principles of Motivational Interviewing, including a patient-centered approach, non-judgmental tone, empathy, support, and avoidance of confrontation. The Video Doctor script and programming avoided the inconsistencies, hesitation, or discomfort that occur all too often in interpersonal encounters. Furthermore, our program circumvented a common barrier to the wider application of MI—the substantial time and training required by health care providers to master it.

**Figure 1:**
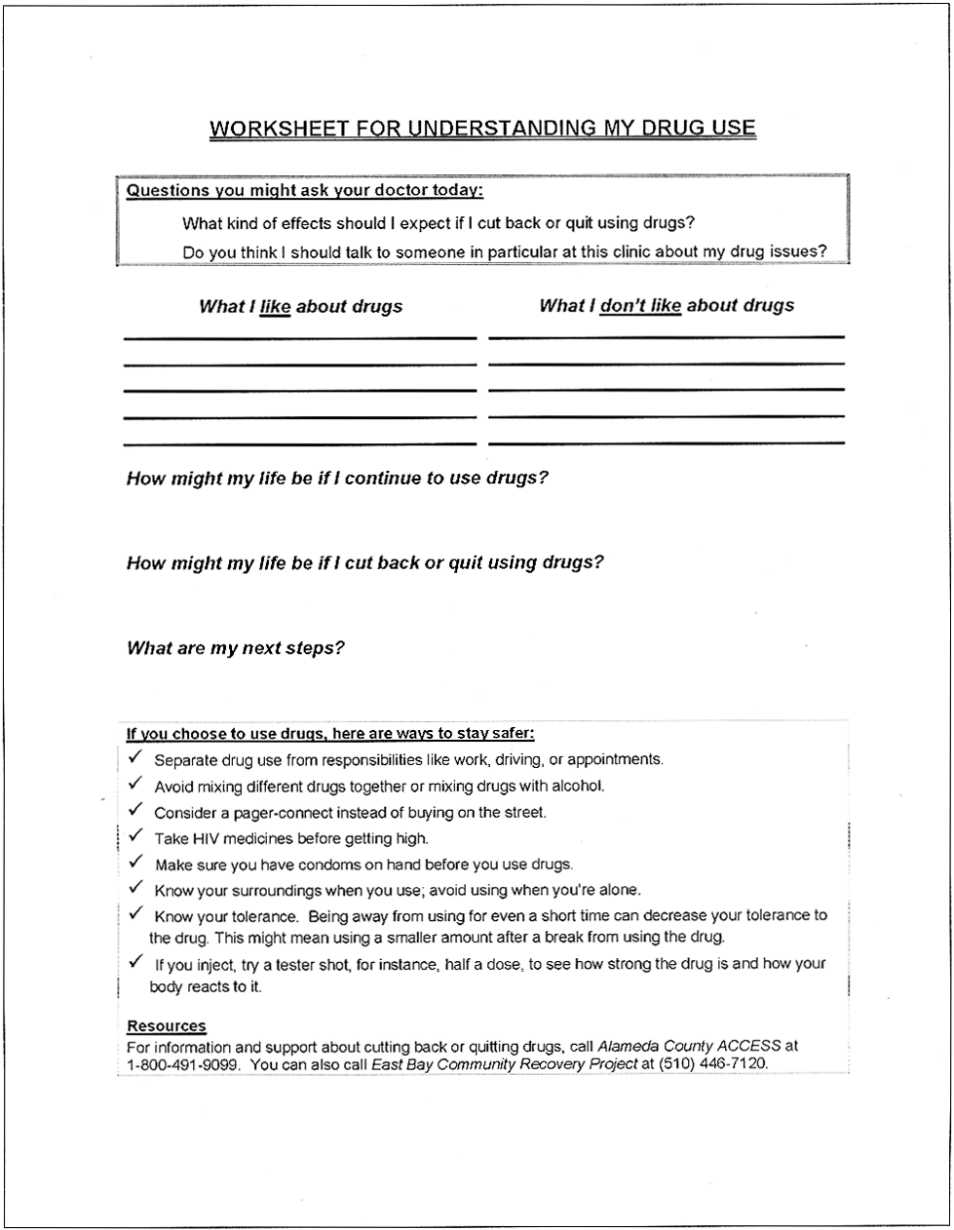
Sample *Positive Choice* Educational Worksheet

**Figure 2:**
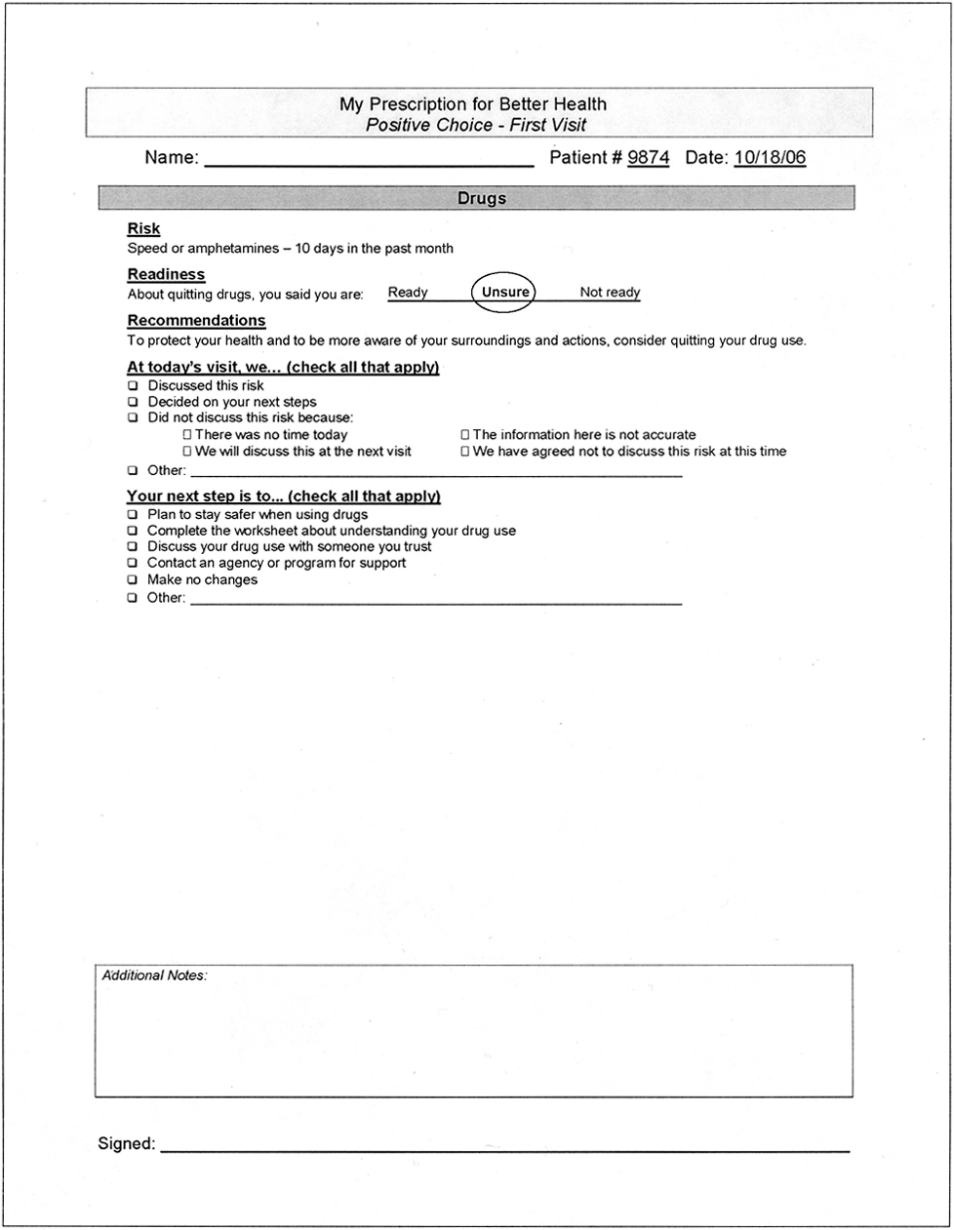
Sample *Positive Choice* Cueing Sheet

Using a library of digital video clips, extensive branching logic, and participant input, the program tailored the video clips to the participant's gender, risk profile, and readiness to change. At the conclusion of each session, the program printed 2 documents: 1) an “Educational Worksheet” for participants with questions for self-reflection, harm reduction tips, and local resources (Figure 1); and 2) a “Cueing Sheet” for providers, which offered an at-a-glance summary of the patient's risk profile and readiness to change, and suggested risk-reduction counseling statements (Figure 2). The Cueing Sheet was discretely placed in the patient's medical record for the provider's use during the medical appointment. Providers were asked to check a box and sign the Cueing Sheet to indicate whether a discussion took place. Intervention participants received “booster” Video Doctor counseling at 3 months, including feedback reflecting changes made since baseline, and updated Cueing Sheets and Educational Worksheets. Sample intervention components, including Video Doctor clips, a Cueing Sheet, and an Educational Worksheet, are available for review on our website, www.ucsf.edu/chips/(new)research-poschoice.htm.

The control group did not interact with the Video Doctor and did not receive the Educational Worksheets or the Cueing Sheets. Following completion of the risk assessment they proceeded to their medical appointment and received the clinic's usual care. Any risk assessment and counseling for the control group was dependent on the medical providers' own initiative and clinical judgment and was not measured.

### Statistical analysis

Baseline characteristics of the intervention and control groups were compared, with p-values obtained by Fisher's exact or chi-square tests. Behavior change outcomes were examined using 3- and 6-month follow-up data for participants reporting risky behavior at baseline and were tabulated by group assignment as binary outcomes (cessation vs. ongoing risky behavior) with Fisher's exact test p-values. Participants could report multiple concurrent risky behaviors, but each outcome was analyzed separately. A Bonferroni correction (α = 0.05/3 = 0.0167) was used to assess statistical significance among the 3 risks with 6-month follow-up the primary time point. For all analyses, we assumed that any participant enrolled in the study who failed to return for follow-up continued their reported risky behavior, constituting a worst-case sensitivity analysis. We also performed alternate outcomes analyses with complete cases only, assuming participants lost to follow-up were similar to those completing follow-ups. Pre-planned sub-group analyses of outcomes were performed by participants' gender, race/ethnicity, educational attainment, hepatitis C co-infection, HIV viral load, source of HIV infection, previous treatment for alcohol or drug abuse, main or casual sex partners, and HIV status of sex partner(s). Univariate summary statistics of mean changes and standard deviations were calculated for specific behaviors in each risk among those completing follow-up. Measures of change included number of drinks per week (for participants drinking over the recommended limit), number of binge drinking episodes, total days of all drug use, absolute percent change in self-reported condom use with main and casual partners, and number of casual sex partners. Differences between groups were compared with t-test p-values. All analyses were done on SAS version 9.1 statistical software (SAS Corporation, Cary NC, USA).

## Results

### Description of sample

**Figure 3:**
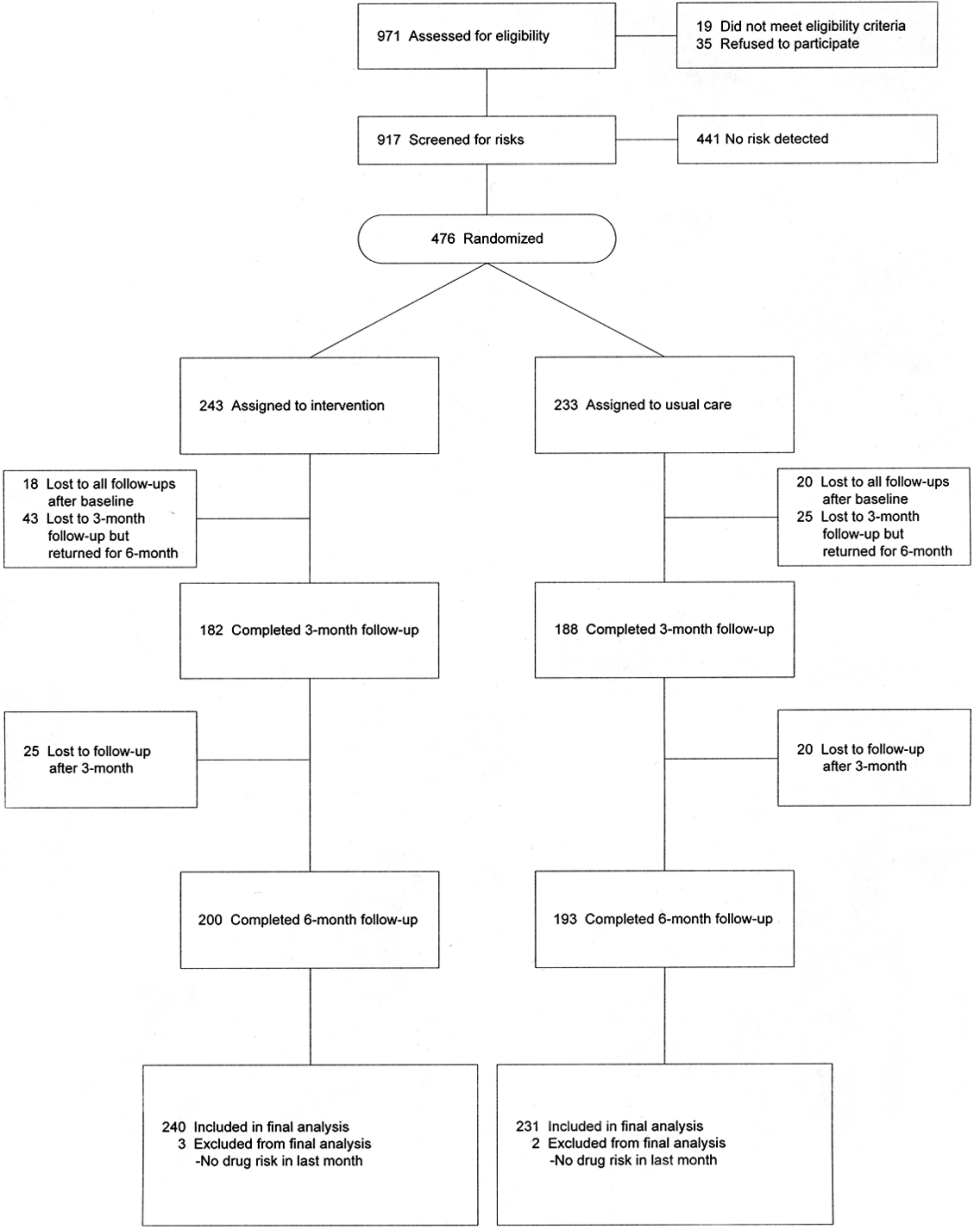
Flow of *Positive Choice* study participants

We invited 971 patients to participate in *Positive Choice*; 19 (2%) failed to meet eligibility criteria and 35 (4%) refused to participate. The remaining 917 patients met eligibility criteria and completed a baseline risk assessment (Figure 3). Of these, 476 participants (52%) reported 1 or more risky behaviors and were stratified by risk combination, then randomized. Five participants reported illicit drug use as their only risk but not within the previous month; we excluded these 5 participants, resulting in a sample of 471 participants for analysis. We achieved high retention for follow-ups; 82% percent of the intervention group and 83% of the control group completed 6-month sessions. Our sample was middle aged, the majority male, and racially/ethnically diverse (Table 1). Most participants had at least a high school diploma. There were no significant differences in demographics or clinical variables between intervention and control groups. There were more participants who exceeded the recommended number of drinks per week in the intervention group than control (51 vs. 37, p = 0.050).

**Table 1:** Demographic and clinical characteristics of Positive Choice sample included in analysis

Variable	Intervention (n = 240)	Control (n = 231)	*P*-value
Age, mean (SD), y	43.9 ( 9.2 )	44.3 ( 9.0 )	0.604
**Gender, n (%)**			
Female	56 (23)	45 (19)	0.315
Male	184 (77)	186 (81)	
**Race/Ethnicity, n (%)**			
Hispanic/Latino	39 (16)	20 (9)	0.088
Black or African-American	118 (49)	118 (51)	
White	65 (27)	72 (31)	
Other or multiple races	18 (8)	21 (9)	
**Educational Attainment, n (%)**			
Less than high school diploma	47 (20)	38 (16)	0.470
High school diploma or GED	139 (58)	127 (55)	
College degree	34 (14)	42 (18)	
Graduate or professional degree	20 (8)	24 (10)	
**Transmission category, n (%)**			
MSM or MSM/W *	122 (51)	119 (51)	0.588
Other sexual risk	55 (23)	46 (20)	
Injecting drug use alone	19 (8)	25 (11)	
Injecting drug use & other risk(s)	13 (5)	19 (8)	
Blood transfusion or blood products	5 (2)	2 (1)	
Multiple Risks	9 (4)	6 (3)	
Don't Know or other	17 (7)	14 (6)	
**HIV Viral Load, n (%)**			
Undetectable	111 (46)	102 (44)	0.716
≤10,000 copies	45 (19)	55 (24)	
10,001–50,000 copies	27 (11)	25 (11)	
>50,000 copies	14 (6)	14 (6)	
Don't know	43 (18)	35 (15)	
**Hepatitis-C Co-Infection, n (%)**			
No	174 (72)	147 (64)	0.103
Yes	46 (19)	62 (27)	
Don't know	20 (8)	22 (9)	
**Risky behavior,** ** ** n (%)**			
Any drug use	105 (44)	95 (41)	0.565
Crack cocaine use	60 (25)	54 (23)	0.681
Methamphetamine use	31 (13)	41 (18)	0.145
Any risky drinking	92 (38)	90 (39)	0.89
Over the recommended limit	51 (21)	37 (16)	0.050
≥3 binge drinking episodes	84 (35)	84 (36)	0.757
Any unprotected sex	143 (60)	141 (61)	0.747
With main partner	99 (41)	90 (39)	0.612
With casual partner(s)	74 (31)	84 (36)	0.204

* *Men who have sex with men (MSM), or men who have sex with men and women (MSM/W)*

** *Not mutually exclusive; participants could report multiple risky behaviors*

Illicit drug use was reported by 200 participants (42% of sample for analysis). Stimulants were the most frequently used drugs (crack cocaine n = 114; methamphetamine n = 72). Risky drinking was reported by 182 participants (39% of sample for analysis), the majority of whom (n = 168) were at risk for excessive binge drinking episodes. Unprotected anal or vaginal intercourse was reported by 284 participants (60% of sample for analysis), and occurred most frequently with main partners only (n = 126), followed by casual partners only (n = 95), and both main and casual partners (n = 63). These risky behaviors were not mutually exclusive; 288 participants reported 1 risky behavior (61% of sample for analysis), while 151 (32%) reported 2 risks, and 32 (7%) reported all 3 risks.

### Process measures

The average baseline risk assessment lasted 19 minutes for participants without any risky behaviors, and 23 minutes for intervention and control participants. The Video Doctor counseling for intervention participants averaged 24 minutes in length across all risk combinations. After the medical appointment and at the conclusion of all study activities, research assistants administered a 4-item acceptability interview to assess participants' reactions to the program. The majority of responses were positive: 892 (97%) “liked” the program or “liked [it] very much,” and 849 (93%) reported it was easy to use. Only 118 (13%) stated the program was “too long,” and only 35 (4%) reported wanting more privacy when using the computer.

Seventy-four percent (181/243) of baseline Cueing Sheets and 72% (131/182) of 3-month follow-up Cueing Sheets were checked or signed by providers, indicating they were used in the medical appointment. Researchers were unable to mandate that providers use Cueing Sheets, and when providers did not use the Cueing Sheets, they often reported deferring their use because of medical contingencies in the appointment.

### Behavior change outcomes

We compared binary outcomes (cessation vs. any ongoing risky behavior) for illicit drug use, risky drinking, and unprotected anal or vaginal sex among intervention and control participants who reported the corresponding risk at baseline. We report proportions of continued drug, alcohol, and sexual risk behaviors in Table 2. Our assumption that participants lost to follow-up continued the risky behavior yielded a conservative estimate of intervention effects; we also present alternate outcomes where those lost to follow-up were excluded; censoring participants lost to follow-up did not substantively change outcomes. We report mean changes and standard deviations for specific risky behaviors among only those completing follow-ups in Table 3.

**Table 2:** Ongoing risky behavior at 3 and 6 months among Positive Choice participants reporting the behavior at baseline

Worst-case sensitivity analysis (assumes ongoing risk for those lost to follow-up)				
Risky Behavior	Intervention	Control	Relative Risk	*P*-value
	n/N (%)	n/N (%)	(95% CI)	
**Any drug use**				
3 months	70/105 (67)	78/95 (82)	0.81 (0.689, 0.957)	0.014*
6 months	59/105 (56)	82/95 (86)	0.65 (0.540, 0.785)	<0.001*
**Any risky drinking**				
3 months	48/92 (52)	56/90 (62)	0.84 (0.651, 1.080)	0.172
6 months	47/92 (51)	53/90 (59)	0.87 (0.666, 1.130)	0.291
**Any unprotected sex**				
3 months	104/143 (73)	117/141 (83)	0.88 (0.773, 0.993)	0.039
6 months	88/143 (62)	108/141 (77)	0.80 (0.686, 0.941)	0.007*

Alternate analysis (excludes participants lost to follow-up)				
**Any drug use**				
3 months	47/82 (57)	50/67 (75)	0.77 (0.608, 0.970)	0.029
6 months	39/85 (46)	60/73 (82)	0.56 (0.433, 0.720)	<0.001*
**Any risky drinking**				
3 months	36/80 (45)	36/70 (51)	0.88 (0.628, 1.220)	0.432
6 months	34/79 (43)	38/75 (51)	0.85 (0.606, 1.191)	0.343
**Any unprotected sex**				
3 months	61/103 (62)	91/115 (79)	0.79 (0.657, 0.938)	0.006*
6 months	67/122 (55)	85/118 (72)	0.76 (0.627, 0.928)	0.006*

* *Significant with Bonferroni correction (α = 0.05/3 = 0.0167)*

**Table 3:** Summary measures of change at 3 and 6 months among Positive Choice participants completing a follow-up session *

Measure of behavior change	N	Mean (SD)	N	Mean (SD)	*P*-value
**Days of drug use in past 30 days**					
3 months	82	−4.0 (11.8)	67	−1.3 (21.2)	0.346
6 months	85	−4.7 (11.6)	73	−0.7 (19.7)	0.130
**Binge drinking episodes in past 3 months**					
3 months	72	−4.2 (7.0)	66	−2.9 (7.3)	0.265
6 months	71	−3.6 (14.5)	69	−3.8 (11.1)	0.899
**Number of drinks per week**					
3 months	43	−9.7 (12.6)	25	−8.1 (18.7)	0.703
6 months	45	−12.7 (13.6)	31	−13.7 (14.9)	0.750
**Absolute percent change in condom use with main partners**					
3 months	75	+0.3 (0.5)	73	+0.2 (0.4)	0.327
6 months	84	+0.4 (0.5)	77	+0.2 (0.5)	0.091
**Absolute percent change in condom use with casual partners**					
3 months	53	+0.3 (0.5)	66	+0.3 (0.5)	0.707
6 months	63	+0.3 (0.5)	68	+0.3 (0.5)	0.858
**Number of casual sex partners**					
3 months	78	−2.3 (9.2)	88	−1.4 (7.9)	0.461
6 months	89	−2.7 (8.4)	93	−0.6 (5.6)	0.042

* *Excludes participants lost to follow-up*

#### Illicit drug use

The intervention group was significantly less likely than the control group to report any ongoing drug use at 3 months (67% vs. 82%, RR 0.81, 95% CI: 0.689, 0.957, p = 0.014). At 6 months, even fewer intervention participants continued any drug use compared with control (56% vs. 86%, RR 0.65, 95% CI: 0.540, 0.785, p<0.001). Univariate measures of change at 3-month follow-up showed that total days of any drug use in the previous month was reduced by a mean of 4.0 days (SD 11.8 days) in the intervention group and 1.3 days (SD 21.2 days) in control (p = 0.346). At 6 months, the intervention group's mean reduction of any drug use was 4.7 days (SD 11.6 days) compared with the control group's mean reduction of 0.7 days (SD 19.7 days) (p = 0.130).

#### Risky drinking

At 3- and 6-month follow-ups, both intervention and control groups showed no significant differences in cessation of risky drinking (52% vs. 62%, RR 0.84, 95% CI: 0.651, 1.080, p = 0.172 at 3 months; and 51% vs. 59%, RR 0.87, 95% CI: 0.666, 1.130, p = 0.291 at 6 months). Looking at univariate measures of change, both groups showed similar reductions of binge drinking episodes and number of drinks per week (for participants drinking over the recommended limit) at both follow-ups.

#### Sexual risks

At 3 months, both intervention and control groups reported less ongoing unprotected anal or vaginal intercourse (73% vs. 83%, RR 0.88, 95% CI: 0.773, 0.993, p = 0.039), however the result did not meet the level of significance set by the Bonferroni correction (p<0.0167). At 6 months, we found a statistically significant reduction of ongoing unprotected sex in the intervention group compared with control (62% vs. 77%, RR 0.80, 95% CI: 0.686, 0.941, p = 0.007). Condom use with main and casual partners was increased by similar modest proportions in both intervention and control groups at both follow-ups. The intervention group showed greater reductions in mean number of casual sex partners at 3 and 6 months compared to control (−2.3 vs. −1.4, p = 0.461, at 3 months; and −2.7 vs. −0.6, p = 0.042, at 6 months).

#### Sub-group analyses

Preplanned sub-group analyses by participants' gender, race/ethnicity, hepatitis-C co-infection, HIV viral load, source of HIV infection, or sex partners' HIV status did not differ from aggregate results. Interestingly, participants' previous treatment for alcohol or drug abuse did not affect substance use outcomes.

#### Adverse events

Because of the sensitive and potentially stigmatizing nature of substance use and sexual behavior, the study team instituted a Data Safety and Monitoring Plan for adverse events, specifically for breach of confidentiality and participants' emotional distress. There were 2 incidents of perceived breaches of confidentiality resulting from participants who did not recall that providers would be given the Cueing Sheet stating their self-reported risky behaviors; both incidents were successfully resolved and the 2 participants continued in the study. Five study participants died during the data collection period. Investigations found that all deaths were attributable to HIV disease and were not associated with participation in the trial.

## Discussion

The *Positive Choice* program identified a high prevalence of ongoing risky behaviors among HIV-positive adults in medical care and achieved significant cessation of illicit drug use and unprotected sex at the group-level, and modest individual-level reductions in days of ongoing drug use and number of casual sex partners compared to the control group. The large trial sample included several important US sub-groups—African-Americans (50% of sample), men who have sex with men (51%), and women (21%)—that are representative of urban, HIV-positive adults in care in the US. Losses to follow-up were low and balanced by randomization group.

*Positive Choice* may be an important adjunct to risk-reduction efforts in routine clinical practice. It incorporates or exceeds all criteria for a best-evidence HIV behavioral intervention as determined by the CDC's HIV/AIDS Prevention Research Synthesis Team, such as prospective design, random assignment to study arms, at least 3 months of follow-up, and at least 70% retention for follow-up [32]. *Positive Choice* extends previous efforts since behavioral interventions for HIV prevention have traditionally focused on sexual behaviors. A review of the recent literature (2000–2004) found only 3 out of 18 (17%) best-evidence behavioral interventions attempted to reduce drug use, either alone or combined with sexual risks [27]. Drug and alcohol use are associated with increased risky sexual behaviors and present additional barriers to optimal medical care, reinforcing the need for integrated risk-reduction strategies. *Positive Choice* addresses multiple risky behaviors and frames these behaviors as a hazard to the patient's own health, in addition to HIV transmission. *Positive Choice* also illustrates a key public health principle—that modest changes at the individual level may actually achieve significant effects at the group level. An additional advantage of *Positive Choice* is its brevity, requiring less than an hour of patients' time for each session before the medical appointment and no continuing medical education for providers given the ease of use of the Cueing Sheets.

HIV care providers' usual screening for and counseling about risky behaviors may be impeded by concerns about stigmatizing the patient, jeopardizing trust between patient and provider, or ethical issues, such as the “duty to warn” HIV-negative sex partners [17]. It is not surprising that a recent survey found overall low rates of transmission prevention counseling to both newly diagnosed and established HIV-positive patients [28]. The *Positive Choice* program successfully overcame providers' traditional barriers to consistent risk screening and counseling by conducting the risk assessment and delivering tailored risk-reduction counseling prior to the medical appointment. Because health risk information was shared with the provider in real time via the Cueing Sheet, the program supported the patient-provider relationship for improved disease management and risk-reduction counseling. An intervention that focused solely on the threat of HIV transmission would have likely brought up patient and provider resistance. By framing risky behaviors as a concern for the patient's own health as well as transmission to others, *Positive Choice* reduced potential stigma and avoided conceptualizing the individual simply as a vector of disease.

We believe that these findings are generalizable to other urban, diverse, English-speaking populations in the US. We make no claims, however, about other settings. Given the worldwide HIV epidemic, future research should explore the efficacy of *Positive Choice* in other languages, sociocultural contexts, or populations. While our findings are limited to risk reduction in HIV-positive individuals, similar strategies may be effective at promoting behavior change in populations at risk for HIV, as well as assessing and counseling persons suffering from other chronic conditions such as diabetes or hypertension, and their underlying risk factors, such as nutrition, physical activity, and smoking.

We recognize several possible limitations. First, there may have been differential disclosure of sensitive behaviors. This bias may have been further enhanced by the possible reporting of risky behaviors to providers through Cueing Sheets. We are unable to validate participants' self-reports, but remain convinced that computerized questionnaires yield more disclosure of sensitive topics than do other, traditional methods, such as face-to-face interviews or paper questionnaires [33], [34]. Although our previous research found that sharing risk information with providers did not inhibit patients' disclosure of sensitive behaviors [33], we have no evidence to deny this occurring in the present study. Second, our risk assessment questions did not assess contextual sexual risk-reduction strategies, such as serosorting (seeking sex only with other HIV-positive people) or strategic positioning (adopting a receptive role with HIV-negative or unknown status partners) [35], [36]. Our trial emphasized outcomes that are reframed as potential acquisition risks to the participant (e.g. new sexually transmitted infections) rather than HIV transmission risks to partners. Future studies may explore these contextual aspects of partner selection and related sexual risk-reduction strategies. Third, we failed to find a significant intervention effect for alcohol risk and speculate that risky drinking might differ from other risky behaviors. Given equivalent reductions by the control group, risky drinking may be particularly responsive to repeated self-assessments. Alternatively, our intervention might have been too brief or the *Positive Choice* Video Doctor model might not be well suited to address risky drinking. Further complicating the topic, alcohol use is legal, socially sanctioned, and may be consumed appropriately. Perhaps risky drinking is more difficult to define than other risks. We recommend that future studies explore differential intervention effects by type of risk. Fourth, absolute risk behavior declined over time among all participants. For example, the proportion of each control group reporting cessation of risk ranged from 14% for any drug use at 6 months (1-[82/95] = 0.137) to 41% for any risky drinking at 6 months (1-[53/90] = 0.411). These declines are consistent with results from other randomized controlled trials of behavioral interventions with HIV-positive individuals [37], [38] and may be explained by the Hawthorne effect, i.e., participants reported declines because they knew they were being studied. Alternatively, declines may have resulted from regression toward the mean, i.e., participants exceeding risk thresholds at enrollment were above their individual averages and fell back to or below their averages at follow-up. The Hawthorne effect in particular is well known in HIV prevention research [39], [40]. This phenomenon, considered a by-product of repeated self-assessments of behavior, is disparately regarded as a major challenge to internal validity [41] or as a potential intervention in itself [42]. Our findings illustrate the complexities inherent in understanding and changing human behaviors. Finally, the discrete contribution of each of the 3 intervention components—Video Doctor counseling, patient Educational Worksheet, or provider Cueing Sheet—remains unknown and worthy of future study.

Responding to calls for “prevention with positives” interventions, the *Positive Choice* program was designed to support medical providers' risk-reduction efforts with minimal additional burden. *Positive Choice*'*s* Video Doctor counseling, patient Educational Worksheet, and provider Cueing Sheet achieved important reductions in risky behaviors among HIV-positive persons. Given the challenges of changing human behavior, our results are notable. *Positive Choice* is an efficacious adjunct to routine medical care for HIV-positive patients with the capacity to have important clinical and public health impact.

## Supporting Information

Checklist S1 CONSORT checklist
(0.06 MB DOC)

Protocol S1 Trial Protocol
(0.05 MB PDF)
