# Supplementary material for: Interactive “Video Doctor” Counseling Reduces Drug and Sexual Risk Behaviors among HIV-Positive Patients in Diverse Outpatient Settings
Source: PLoS One. 2008 Apr 23;3(4):e1988. doi: 10.1371/journal.pone.0001988 (PMC2292251; doi:10.1371/journal.pone.0001988)
Supplement: Protocol S1 — Trial Protocol (0.05 MB PDF) [file pone.0001988.s002.pdf]

1. Study Aim/Purpose

Significant proportions of HIV-positive individuals engage in sexual behaviors that put others at risk for HIV infection. Many patients with HIV also abuse alcohol or other drugs, putting themselves at risk for medical complications and contributing to increased risk of HIV transmission to uninfected partners. We propose to conduct a randomized, controlled study to determine if Positive Choice, an interactive, multimedia program that delivers a brief motivational intervention and cues providers can 1) reduce the frequency of alcohol use, 2) reduce the frequency of hard drug use, 3) reduce the rate of unprotected sex, and 4) increase the rate of disclosure of HIV-positive status to sexual partners compared with a control condition.

As we begin pilot testing our *Positive Choice* study, we propose to conduct a sub-study, with the providers and the first 100 patients enrolled and agree to participate, to help us evaluate the efficacy of our computerized risk assessment program at eliciting patients' risk behavior disclosure and understand how we can maximize such disclosure.

1. For each of the risk behaviors we are examining (alcohol abuse, illicit drug use, unprotected sex, failure to disclose HIV status to sexual partners), how do providers' knowledge, beliefs, and assumptions of patients' risk behaviors compare to risk behaviors patients disclose when completing our computerized assessment?
2. For each of the four risk behaviors, how much underreporting, either to providers or other formal assessment, actually occurs?
3. To what degree do patients' attitudes about their providers' role and/or the social desirability factor or "self-deception" contribute to any underreporting of these risk behaviors?

We propose to survey providers about their existing knowledge of each of the first 100 patients' risks, their sources of information, and their confidence in such knowledge. We also propose to conduct

confidential interviews with each of these 100 patients, following their completion of the computerized assessment and video doctor program.

2. Background

Of the estimated 650,000 to 900,000 people living with HIV in the United States, about two thirds are aware of their seropositive status, and over 70% are sexually active after they learn they have been infected. Recent data indicate an increase in the sexual risk behaviors of both HIV-negative and HIV-positive individuals. Between 1993-1994 and 1996-1997, the prevalence of unprotected anal intercourse rose from 37% to 50% among men ages 18-29 who have sex with men. In a 1999 study, 42% of men and women with HIV reported at least one occasion of unprotected vaginal or anal sex in the preceding 6 months. Unsafe sex also puts HIV-positive persons at risk for contracting other sexually transmitted diseases, thereby endangering their own health. One study found that 15% of individuals recently diagnosed with HIV returned within 3 months with a new STD diagnosis. Although female-to-male transmission of HIV is much less efficient than male-to-female or male-to-male transmission, sexually transmitted diseases, particularly those that ulcerate, are a major co-factor of HIV transmission for both men and women. Because HIV-positive women are less likely than men to know that they have a sexually transmitted disease, it is important that they use condoms consistently to prevent transmission of HIV and other STDs. Together, these data speak to the urgency for developing interventions to reduce sexual risk behaviors among HIV-positive individuals.

These health concerns fall disproportionately on certain populations, which is why the CDC's HIV prevention strategic plan calls for prevention initiatives for diverse populations. We are proposing to situate our study at an HIV outpatient clinic in Alameda County, California. Oakland, the County's

largest city is the second most diverse city in the United States. AIDS is the leading cause of death for males ages 25 to 44 in Alameda County.

3. Significance

Risk-reduction interventions should target HIV-infected individuals whose risk profiles include alcohol and drug use, contextual substance use (intoxication immediately before sex), and/or unsafe sex. Further, these efforts must address the co-morbid problem of substance abuse and other major psychological determinants of unsafe sex among HIV-positives: 1) believing that taking combination therapy and/or having an undetectable viral load protects sexual partners from contracting HIV; 2) having less concern about HIV due to the availability of new treatments; 3) attributing responsibility for protection to one's partner; 4) avoiding the use of behavioral coping strategies; and 5) having negative attitudes about condoms.

Multimedia technology has great promise for enhancing physicians' prevention efforts by delivering brief motivational interventions to at-risk, under-treated populations. Audio-enhanced video reduces demand for high literacy and enhances information retention. These programs could ensure that all patients receive basic risk-reduction messages, and, by producing a cueing sheet, they could allow time-pressured providers an individualized prompt to reinforce key risk-reduction messages. We propose to develop *Positive Choice,* a multimedia program featuring an audio-visual computer assessment of patients' substance use and sexual risks followed by a video doctor who delivers a brief (20-minute) motivational intervention (based upon motivational interviewing), which is tailored to patients' readiness to change and risk profile. Providers will receive education in how to use the cueing sheets to reinforce program behavior change goals. The *Positive Choice* intervention can be seamlessly integrated into primary care for HIV-infected individuals. In the proposed randomized, controlled trial,

patients at a public hospital-based HIV-outpatient clinic will complete the *Positive Choice* multimedia program immediately before their medical appointment.

We recognize that substance use and risky sex are complex problems, not easily modified without meaningful effort. We doubt that our intervention alone would be powerful enough to eliminate these risk behaviors altogether. However, when combined with provider-directed intervention, *Positive Choice* could help significantly reduce these serious health risks.

Risky health-related behaviors, such as alcohol misuse, illicit drug use, unprotected sex, or failure to disclose HIV status to sexual partners, are sensitive topics, the disclosure of which may be stigmatizing to a given patient or research subject. Reasonably accurate screening for these risks is, however, a procedural foundation of our *Positive Choice* intervention study with HIV-positive patients. Failure of patients to disclose such behaviors could result in our failure to recruit adequate numbers of subjects or our inability to detect potential effects of our video doctor intervention. Health care providers at our upcoming study sites and clinician collaborators have suggested that their patients underreport these risks, particularly illicit drug use.

Patients' willingness to disclose health-risk behaviors to health care providers may be related to their comfort with answering screening questions and their perception of how relevant these inquiries are to their health care. It is unclear whether or not patients are more willing to disclose risk behaviors if they know the information will not be passed along to their providers. In an earlier study we conducted, it made no difference in primary care patients' disclosure of HIV, alcohol, drug, or domestic violence risks whether they were told their providers would or would not be informed of their responses. Yet in personal communications with several clinician collaborators, we have been advised that patients would disclose more often if they thought their providers would not have access to the information. Another factor that may suppress full disclosure was suggested by one of our collaborators, who is also a

provider at one of our *Positive Choice* study sites. It is her impression that patients often fail to disclose sensitive or stigmatizing behaviors, not because they consciously underreport, but because their own self-perception distorts their assessment of the existence or degree of such behaviors.

Our proposed sub-study will be important for several reasons. First, as we pilot *Positive* Choice, it will be important to assess its acceptability to patients and to be able to compare our computerized risk assessment with the current “standard” of risk assessment providers use at our study sites. Second, it could make a significant difference in risk disclosure if patients are unwilling to disclose that information to their providers. We have not replicated the finding from our earlier study that patients do not disclose differently depending on whether or not their provider has access to their answers, and we are unaware of other studies addressing this question. Third, on exploring the literature, it appears those items in a psychological measure of “social desirability” measuring “self-deception,” as developed by Paulhus (1984), and currently in use, including with HIV risk behavior (Latkin 1998), may best reflect the self-deception that we propose may inhibit full disclosure. If we measure our sub-study patients on this factor, we will be able to check for an association between self-deception and degree of risk reported, which will give us an indication of the degree to which self-deception distorts patient self-reports.

4. Methods

4.A. Overview of Design

We expect to recruit 800 patients to participate in the initial risk assessment. The computer program will assess HIV-positive patients’ substance use and sexual risks. During this same session, the computer program will randomly assign eligible patients (n=512) to an Intervention or a Control Group. Intervention Group patients (n=256) will receive the *Positive Choice* intervention, which employs

principles of motivational interviewing, to help motivate them to reduce their substance use and sexual risk behaviors. The *Positive Choice* program will automatically produce a provider cueing sheet for Intervention Group participants, summarizing their substance use and HIV transmission risks and suggesting risk-reduction messages, which will be placed in their medical record. Control Group patients (n=256) will only receive the computer risk assessment and will otherwise receive the usual care offered to HIV-positive individuals in these settings. Immediately following the initial computer session, patients will complete their scheduled clinic visit. To ensure that providers at the study site can effectively incorporate information contained in the cueing sheets into the patient visit, they will complete a skills-based course and receive academic detailing during the study period.

Three months after the baseline assessment and intervention, Intervention and Control Group patients will complete an additional assessment. The Intervention Group will also receive a *Positive Choice* "booster" intervention. Six months after baseline, both groups will complete a final assessment.

We will examine group differences over time in 1) the frequency of alcohol use; 2) the frequency of drug use; 3) the proportion of vaginal or anal sex without a condom; and 4) the rate of disclosure of HIV-positive status to sexual partners. We also will examine the effect of the intervention on patients' readiness to address substance use and sexual risk behaviors.

For our sub-study, during the first few weeks of our pilot study, at the beginning of each week, we will administer a written survey to each provider about his or her knowledge of the risk behaviors of each patient he or she will see that week. Providers will be asked about each of the four behavioral risks for each patient, the source of any knowledge they may have, and the confidence in their knowledge about that patients' risk behaviors. [See Appendix H.]

In addition, following their computerized risk assessment (and video doctor advice, if applicable), each patient will be asked if he or she would be willing to complete some additional information in order to help us know how we can best ask patients about private behaviors. If the patient agrees, he or she will be assured that for this interview, his or her responses will be confidential and his or her provider will not be given the information. The interview will include questions about (1) the patient's comfort and opinion about the computerized risk assessment he/she had just completed, (2) the patient's opinion about which method of risk assessment would make him or her most comfortable answering sensitive questions honestly, (3) the patient's attitude about the appropriateness of asking about these risks in the health care setting, (4) any concerns about confidentiality, (5) any concerns about their answers to the computerized risk assessment. Questions will be asked in an open-ended form. The research assistant will also ask the patient 20 questions (constituting the measure of "self-deceptive positivity") from the Balanced Inventory of Desirable Responding (BIDR) (Paulhus, 1988). The questions ask patient to respond on a scale of 1 to 7, depending on whether the patient considers to item to be "not true" ranging to "very true." [See Appendix H.]

We will examine (1) differences between the providers' risk assessments and the *Positive Choice* computerized risk assessment results for these 100 patients; (2) patients' attitudes about the acceptability of the *Positive Choice* risk assessment; (3) a possible association between "self-deception" scores and risks reported in the *Positive Choice* assessment; (4) patients' concerns about confidentiality of the *Positive Choice* risk assessment and differences between risks reported there and in a confidential interview without the providers being given the information.

4. B. Methods of Data Analysis

We will first describe the sociodemographic characteristics of our sample, as well as provide descriptive statistics for the measures of risk (e.g., substance use, frequency of unsafe sex). Given that

the majority of our outcome variables are dichotomous or ordered categorical variables, we will use standard or ordinal logistic regression methods, respectively, to describe associations and test specific hypotheses among the variables. Analyses will be required for the following:

-- To compare rates of alcohol use and drug use for the intervention versus control groups over the course of the entire study. These data will be modeled as a function of relevant baseline covariates. To allow for the multiple assessments of risk over time, we will use the generalized estimating equation approach or a generalized linear mixed-model approach.

-- To compare rates of reported unprotected sexual intercourse and rates of disclosure of HIV status to sexual partners for intervention versus control group over the entire study period. As above, we will take the inherent dependence of these data into consideration in the analysis using the logistic regression model to assess the effect of the intervention. The logistic model will include factors for the study interventions, possible confounding variables, and demographic information.

For our sub-study, we will provide descriptive statistics of our measures of acceptability: patients' attitudes and opinions about the computerized risk assessment they have completed, concerns about confidentiality and providers' knowledge of reported risk behaviors, and degree to which patients increase risk behaviors they report when they know providers will not be given the information. For each of the four risk areas we will use chi-square tests to examine the association between each risk outcome and assessment method: providers' usual risk assessments and the *Positive Choice* computerized assessments. We will similarly assess the associations between "high" versus "low" "self-deception" scores and (1) overall level of risk behaviors and (2) level of drug risk behaviors assessed by *Positive Choice.* Because some of the questions about patients' opinions of the risk assessment process will be open-ended, we will also be obtaining qualitative data.

4. C. Subject Selection

1. Who and Why.

Providers. The study site is staffed by 10 physicians and nurse practitioners, all of whom will be invited to participate in the study.

Patients. We will recruit HIV/AIDS patients from two outpatient medical clinics that serve an ethnically diverse and economically disadvantaged population. This population is in significant need of HIV transmission prevention interventions, as noted by the CDC.

2. Total Number/Number per Group.

Providers. We will invite 10 providers (physicians, nurse practitioners) to participate and anticipate that all of those invited will agree to participate.

Patients. We will recruit 800 patients for the initial risk assessment; 512 with alcohol and/or drug and/or sexual transmission risks will be randomly assigned to the *Positive Choice* intervention (n = 256) or the control group (n = 256).

For our sub-study, we will invite all patients who have completed the initial risk assessment to participate in our additional interview, until we obtain 100 participants.

3. Inclusion/Exclusion Criteria.

Providers. To participate, the provider must provide care to HIV/AIDS patients at the study site.

Patients. To participate in the initial risk assessment, participants must be 18 years of age or older, HIV-positive for three months or more, a patient at the clinic coming for a follow-up visit, and able to understand English. To be eligible for randomization, participants must report having alcohol use, drug use, and/or sexual transmission or disclosure risks. The study will include women and members of minority groups. Individuals between 18 and 21 will be included because this age group is represented in the patient population at the study site. Children less than 18 years of age will not be included, because they are not treated at the site.

4. D. Subject Recruitment

4.D.1. Sources

Provider recruitment. The medical director for the study site will provide a list of physicians and nurse practitioners who provide care at the site.

Patient recruitment. More than 1,000 HIV-positive patients receive care at the study site (see Appendix B, letter of support). Most patients come into the clinic four to five times per year. To recruit 800 eligible patients into the study, we estimate that we will need to invite approximately 1,000 patients to participate. Based upon our prior work with this population, we expect no more than 20% to decline to participate or be excluded due to lack of English comprehension, cognitive impairment, or other practical barriers. The remaining 800 patients will participate in a baseline risk assessment. Of those, we expect that 64% (n=512) will be at risk and will enroll in the intervention study.

4.D.2. Initial Contact Method

Provider Recruitment. We will send providers letters of invitation, signed by the site's medical director and by Dr. Gerbert, describing the study, explaining its joint sponsorship by their institution, and informing them of the risks and benefits of participation and the potential benefits to their patients. Providers who are interested in the project will mail a return form to Dr. Gerbert at UCSF. Upon receipt of the form, Dr. Gerbert will call the interested provider, further describe the study, and answer questions. If a response postcard is not returned within two weeks, Dr. Gerbert will place a follow-up telephone call to the providers who did not respond. At that time, interested providers will receive detailed information about the study and will have the opportunity to ask questions. Dr. Gerbert has successfully used similar recruitment protocols in previous studies. See Appendix C for the provider invitation letter.

Patient Recruitment. Patient recruitment will be integrated into the normal operations of the study site. Large posters describing the study will be posted in the clinic waiting room at least two months before beginning participant recruitment (see Appendix D, recruitment posters). The posters will announce the start date of the study, describe the study's goals, eligibility criteria for participating in the screening, explain the time that participation requires, and the need to arrive an hour before one's scheduled appointment, and to approach the research assistant in order to participate. Once the study begins, we will place new posters in the waiting room that contain all the information listed above and invite the patients to ask the research assistant for more information if they are interested in the study. We will also work in conjunction with the staff at the clinic to have them hand out informational flyers to patients when they are checking in for their appointment. Patients who are interested in participating but do not arrive sufficiently early may approach the research assistant. In these situations, the patients will be invited to participate in the study beginning with their next scheduled appointment. Patients who "drop in" (arrive without scheduled appointments) and are interested in participating will be directed to a research assistant after checking in. These patients will be invited to immediately enroll and participate in the study. A pilot survey of Highland patients in the clinic waiting rooms revealed that a high percentage (85%) would be sufficiently motivated by the offer of a $40 incentive to come early to their appointment to participate. We have discussed the proposed procedures with clinic staff to ensure that patients' privacy is optimized.

For our sub-study, all patients who have completed their initial risk assessment (and the video doctor session for the intervention group subjects) will also be asked if they would be willing to answer some additional questions to help us evaluate our *Positive Choice* risk assessment. We will recruit until we obtain 100 patients.

4. E. Consent Process and Documentation

Provider Consent Process. If interested in the study, providers will be asked to complete a consent form prior to participation. See Appendix A for the provider informed consent document.

Patient Consent Process. Two research assistants will be working at each study site, one located in the waiting room and the other in a nearby private room. The research assistant in the waiting room will work with the clinic staff to determine each arriving patient's status as related to the study (e.g., arrived early to participate, drop in, declined previous invitation, etc.). Patients who are interested in the study and who approach the research assistant will be directed to the second research assistant located in the private room. This research assistant will be responsible for obtaining informed consent, obtaining contact information, giving cash reimbursements, and delivering the provider cueing sheets to clinic staff to post in the patient's chart. Patients will be informed that participation involves completing a health history assessment which may provide health information and recommendations, and that their responses might be shared with their clinic provider. At the end of the study, we will debrief each subject and describe the components of the Intervention and Control Groups. See Appendix A for the patient informed consent document.

Patients who agree to participate in the proposed sub-study will be asked to complete an additional consent form (see Appendix G.).

4. F. Procedures

1. Study Procedures.

Provider Survey and Skills-Based Training. Providers who enroll in the study will complete a brief (half hour) face-to-face interview with a UCSF research assistant at two time points throughout the duration of the study. These interviews will assess providers' attitudes and behaviors about screening patients for behavioral risks. Providers will also complete a one-hour skills-based course. This training session will address many of the barriers that keep providers from counseling patients about prevention

as well as provide an orientation to the *Positive Choice* Intervention. To ensure that providers are appropriately implementing the cueing sheets, project staff will conduct brief academic detailing sessions over the duration of the project.

Initial Assessment. A research assistant will escort the patient to a nearby private room containing computer stations. Patients' privacy will be ensured through the design of the kiosk and use of headphones. A research assistant stationed in the room will input the patient's ID number and launch the program. After making sure the participant is able to hear the instructions over the headphones, the research assistant will allow him or her full privacy. Although the computer assessment will be designed to be self-administered, the research assistant will be available to help. The patient will then proceed to a series of demographic questions. The computer assessment will use audio as well as visual (displaying the text of the question and response options on the screen) methods to ask participants about their risks. Participants respond by using a simplified, easy-to-use, color-coded keyboard. Although the risk assessment collects sensitive information, it will not collect information that might indicate an imminent threat of harm to the patient or others, or any other reportable event such as child abuse. Following risk assessment, patients without risk factors are excluded from further study, thanked for their participation, and given their reimbursement by the research assistant.

For our sub-study, at the providers' training session, they will also be introduced to sub-study, and will be informed that for the first few weeks of the study, they will each receive the weekly survey sheet on their existing assessment of individual patients' risks. (Appendix H.).

Randomization and Intervention. Patients with risk factors will be randomized into either the Intervention or Control Group. Patients randomized to the Intervention Group will immediately receive a brief motivational intervention. The *Positive Choice* intervention will feature risk-reduction messages delivered by an actor-portrayed video doctor, tailored to each participant according to his/her gender,

level of risk, and other factors. The components of the intervention are 1) personalized feedback, 2) tailored advice messages, 3) patient prompt sheet, and 4) provider cueing sheet. The cueing sheet is produced by the program on completion and given to the provider by the research assistant. Patients in the Control Group will not receive an intervention. They will be thanked for their participation and reminded to return for computer assessment in three and six months.

Booster Intervention Session. Patients in the Intervention Group will receive the *Positive Choice* intervention a second time at the 3-month follow-up. Similar to the initial session, the booster session will provide feedback about how the patient's current risk behaviors compare with his/her previous behaviors. Control group patients will not receive a booster intervention.

Evaluation of *Positive Choice Program*

Following their risk assessment or following the video doctor intervention for those assigned to the intervention group, all participants, whether or not reporting risks, and regardless of group assignment, will be asked to complete a brief evaluation of the *Positive Choice* computer program. See Appendix F. for the evaluation questions.

For our sub-study, all participants, until we obtain 100, will then be invited to help us evaluate the program by answering some additional, confidential questions about their views on the program and on the risk assessment process generally. Questions asked for our sub-study are presented in Appendix H.

2. Time.

Providers. The training session will last one hour and the interviews will take a total of one hour (30 minutes/interview). We do not anticipate any increases in amount of time providers spend with their patients.

Patients. The initial assessment will take a maximum of 10 to 15 minutes. The video doctor intervention will take about 10-30 minutes. At the 3-month follow-up clinic appointment, the Intervention Group patients repeat the 10-15 minute assessment and receive a 10-30 minute booster intervention. At the 3-month follow-up clinic appointment, the Control Group patients repeat the 10-15 minute assessment. At the 6-month follow-up clinic appointment, the patients in both groups repeat the 10-15 minute assessment. In total, patients in the Intervention and Control groups will spend 50-105 and 30-45 minutes, respectively.

For our sub-study, patients will spend an additional 10 minutes.

3. Sites. The study sites will be the HIV-outpatient medical clinics at Highland Hospital in Oakland and Fairmont Hospital in San Leandro. These hospitals are part of the county health system for Alameda County in the San Francisco Bay Area. More than 1,000 HIV-positive patients receive outpatient health care at the study site. Please see Appendix B for a letter of support from Kathleen Clanon, MD, Medical Director of Alameda County Medical Center HIV Services.

4.G. Risks/Discomforts

The principal risk for participants in this study is loss of confidentiality. Since questions about sexual behavior and alcohol and drug use are personal, and information about patients' risk status is sensitive, every effort will be made to minimize loss of confidentiality. The computer risk assessment will be conducted in a private room with private kiosks and headphones designed to maximize patients' privacy.

Another risk to patient participants is the potential emotional upset that may accompany feedback about their substance use and/or HIV-transmission risks. The UCSF research assistant will also be available to direct patients to local services for counseling or other interventions as needed. Our

study will take place within the patient's healthcare setting, which can offer support and referrals to any patients who are upset.

Provider participants run no risk of adverse events.

4.H. Treatment and Compensation for Injury N/A

4.I. Alternatives

Providers. Providers who elect not to participate in the study will be thanked and excused.

Patients. Patient subjects who elect not to take part in the study may opt for no treatment or may avail themselves of the resources located within their medical settings or elsewhere. Both facilities offer HIV counseling services, in-house alcohol and drug treatment programs, outpatient psychological counseling services, and health education programs.

4.J. Costs to the Subject

There are no costs to provider or patient subjects for their participation in this study. Some patient subjects will be referred to free alcohol and drug abuse counseling services offered at the medical center.

4.K. Reimbursement of Subjects

Providers. Providers will not receive reimbursement for their participation.

Patients. Patient participants will be reimbursed with a $40 gift certificate to Safeway or Target at baseline. At 3- and 6-month follow-up sessions, the reimbursement rate will increase to $50 and $60 in gift certificates. The total reimbursement available to participants completing all assessments is $150 in gift certificates.

4.L. Confidentiality of Records

Each participating patient will be assigned a unique identification number. Subjects will be identified by their unique identification number only on response sheets and computer data files. All

patient contact information and a master list linking the code numbers with participants' names will be maintained in a secure, locked cabinet in the office of the Principal Investigator. A separate and secure master list linking patient names with their unique identification numbers will be retained so patients can be contacted for follow-up. Signed consent forms will also be kept in a locked file separate from any study data. The provider cueing sheets will have patients' names written on them by the UCSF research assistants, but will immediately be placed in patients' medical records, the security of which will be maintained by the participating clinic. At the end of each day of data collection, the research assistant will copy all data from the computer hard drive to a Zip disk or CD-ROM, and will erase the file stored on the hard drive. Access to the computer hard drive will be password protected. The Zip disks or CD-ROMs will be stored in a locked cabinet. At the end of the study, these research materials will be destroyed. We have also obtained a Certificate of Confidentiality from NIDA to ensure that patients' data, including the provider cueing sheet, cannot be obtained through forced disclosure.

5. Qualifications of Investigators

Dr. Gerbert, the Principal Investigator, has been studying health-promoting behaviors of health care professionals and their patients for nearly two decades. In addition, she is an expert in the measurement of outcomes of health care and health promotion. Dr. Gerbert has been conducting research with HIV-care providers and HIV-infected individuals since 1985.

6. Reference to Special Requirements and Attachments

Appendix A: Consent Forms; Appendix B: Letter of Support; Appendix C: Provider Invitation Letter; Appendix D: Patient Recruitment Posters; Appendix E: Draft Instruments.

7. Bibliography

1. Gerbert B, Bronstone A, Pantilat S, McPhee S, Allerton M, Moe J. When asked, patients tell: Disclosure of sensitive health-risk behaviors. *Med Care*. 1999;37:104-11.
2. Gerbert B, Bronstone A, McPhee S, Pantilat S, Allerton M. Development and testing of an HIV-risk screening instrument for use in health care settings. *Am J Prev Med*. 1998;15:103-13.
3. Gerbert B, Bronstone A, Clanon K, Abercrombie P, Bangsberg D. Combination antiretroviral therapy: Health care providers confront emerging dilemmas. *AIDS Care*. 2000;12:409-21.
4. Gerbert B, Moe J, Saag M, et al. Toward a definition of HIV expertise: A survey of experienced HIV physicians. *AIDS Pat Care STDs*. 2001;15:321-330.
5. Gerbert B, Brown B, Volberding P, et al. Physicians' transmission prevention assessment and counseling practices with their HIV positive patients. *AIDS Educ Prev*. 1999;11:307-20.
6. Gerbert B, Bleecker T, Bernzweig J. Is anybody talking to physicians about acquired immunodeficiency syndrome and sex? A national survey of patients. *Arch Fam Med*. 1993;2:45-51.
7. Gerbert B, Maguire BT, Bleecker T, Coates TJ, McPhee SJ. Primary care physicians and AIDS: Attitudinal and structural barriers to care. *JAMA*. 1991;266:2837-42.
8. Bangsberg D, Tulsky J, Hecht F, Moss A. Protease inhibitors in the homeless. *JAMA*. 1997;278:63-5.
9. Paulhus D. Measurement and control of response bias. In: Robinson J, Shave P, Wrightsman L, eds. *Measures of personality and social psychological attitudes*. New York: Academic Press; 1984:17-59.
